# American Society of Dental Surgeons

**Published:** 1842-03

**Authors:** 


### Miscellaneous Notices.

*Bibliography.*—We have several dental works which it was our intention to have noticed in the present number of the Journal, but are prevented from doing so for want of room; we shall endeavour however, to attend to them in our next.

---

*American Society of Dental Surgeons.*—When the formation of this society was first talked of, the very idea of such an association was hooted at and scouted by many as impracticable. Various objections were raised, and unexpected obstacles thrown in the way. One was unwilling to impart from the stores of knowledge which he gave himself the credit of possessing, to gentlemen of the profession of merely creditable pretensions. "A community of knowledge! oh no," says another, "I can never consent to that!" A third says, "I am no society man," and a fourth objected on the ground that it would be likely to bring him into contact with those whom he supposed occupied a less lofty place in the temple of professional fame, than that to which he had attained. As for being benefitted himself by an interchange of views, the idea was not to be thought of; it was preposterous; he had already drank too deeply from the fountains of knowledge to learn more, and was unwilling to bestow any part of his choice and invaluable treasures upon others. But, the society was formed, and under circumstances as auspicious as any of its warmest advocates had dared to anticipate. It has now been in existence for nearly two years, and we are of those who believe that much good has already resulted from it, and that it is destined to achieve for the profession and community at large, and that too, before many years shall have passed, a greater amount of benefit, than individual effort ever has, or ever could have accomplished. The members of the society may perhaps, therefore, regard it as a fortunate circumstance that they have none of these false prophets among them. Men of such selfish and narrow-minded views ought not to be admitted to membership in the society, and that they have not been, may be considered as a fortunate circumstance. We are happy to believe that among the more scientific and intelligent part of the members of the profession, there are few such, and it is only from this class, that the society should be made up.

It must be a source of gratification to the members of the society to know, that the efforts which they are making to advance the science of dental surgery, and elevate the dignity of the profession, and purge from it the empiricism with which it has so long been infested, meets the hearty and warm approval of the most distinguished of their professional brethren both at home and abroad. We have received letters from many of the most eminent European practitioners, expressive of their high gratification, and proffering for the advancement of the interests of the society, whatever aid it may be in their power to bestow. Thus encouraged, and sustained by the best wishes of the medical profession generally, they have every inducement to go on. The cause in which they are engaged is a good one, and we say to them relax not your efforts in it, but persevere, and you will reap the rich reward of knowing that you have not only added to the respectability and usefulness of your calling, but to the good of your fellow beings.

But, as we have on another occasion said, let us repeat, admit none to fellowship with you, except men of creditable scientific pretensions. You had better offend half a dozen unworthy applicants for membership by rejecting them, than to admit one who is not qualified to practise the profession, and by so doing, bring reproach upon your body. But, while on the one hand, caution is necessary, it should not on the other, be carried so far as to exclude such as are really worthy of a place among you.

The time appointed for the next meeting of the society is rapidly approaching. It is now near at hand, and we hope the members of the society will bear this in mind, and make their arrangements in time to attend. We are anxious that there should be a full meeting, and although the place in which it is to be held, is a little more distant from the residences of southern and western members, than was that of either of the preceding ones, we hope that that circumstance will not prevent any from being present. At the last meeting which was held in Philadelphia, Dr. L. S. Parmly, of Mantanzas; Dr. Ludolph Parmly, of Mobile; Dr. E. Taylor, of Natchez; and G. W. Parmly, of New Orleans; were in attendance. We hope to have the pleasure of greeting them again at our next meeting in Boston, and many of our other southern and western brethren. Every member should be willing to make some sacrifices to aid in the accomplishments of the important objects of the association. Although it was at a sacrifice of considerable time and money, that many who were present at the two preceding meetings, held in the cities of New York and Philadelphia, attended, we do not think that any regretted having done so. Both of these meetings were highly interesting and the attention which the distant members received from those resident in these places, were peculiarly pleasant and gratifying. We do not know how the members of our own immediate profession regard us in Boston—the far famed and celebrated nursery of science; but we doubt not that we shall receive a cordial welcome. The professors of the Mason street Medical College, as we learn by letter from our highly esteemed friend, Dr. Smith, editor of the "Boston Medical and Surgical Journal," have, in a high-minded and praiseworthy spirit of liberality, tendered to the society the use of their principal lecture room. This act of unlooked for courtesy, merits from the members of the society their warmest thanks.

The next meeting is looked forward to with much interest, and we expect from what we have been able to learn, that members from almost every section of the country, will be present.

Dr. Eleazar Parmly, first Vice-president of the society, will deliver the opening address, and from the well known abilities of that gentleman, a rich intellectual treat may be anticipated; dissertations will also be delivered by E. Baker, M. D. of New York; E. Townsend, of Philadelphia; V. Cuylor, M. D. of Hartford, Connecticut; H. M. Hayden, M. D. of Baltimore; and E. Maynard, M. D. of Washington City. Besides these, it is expected that many interesting papers will be read on the occasion.—*Balt. Ed.*

